# Association between COVID-19 pandemic and the suicide rates in Nepal

**DOI:** 10.1371/journal.pone.0262958

**Published:** 2022-01-24

**Authors:** Binod Acharya, Keshab Subedi, Pramod Acharya, Shweta Ghimire

**Affiliations:** 1 Urban Health Collaborative, Drexel University, Philadelphia, Pennsylvania, United States of America; 2 iREACH, ChristianaCare Health Systems, Wilmington, Delaware, United States of America; 3 Public Affairs Reporting, Kathmandu, Nepal; 4 Center for Bioinformatics and Computational Biology, University of Delaware, Newark, Delaware, United States of America; University of Manitoba, CANADA

## Abstract

**Background:**

Past works have linked the COVID-19 pandemic and subsequent public health responses such as isolation, quarantine, and lockdown to increased anxiety, sleep disorders, depressive symptoms, and suicidal ideation. Only a few studies, mostly carried out in high-income countries, have investigated the association between the pandemic and suicide rate. We seek to investigate the changes in the monthly suicide rates during the COVID-19 pandemic in Nepal, compared to the pre-pandemic suicide rates.

**Methods and findings:**

This is a retrospective study investigating the changes in suicide rates in Nepal during the COVID-19 pandemic period (April 2020 to June 2021), compared to the pre-pandemic period (July 2017 to March 2020), adjusted for seasonality and long-term trend in the suicide rate. We performed analysis for the entire country as well as sub-sample analyses stratified by gender and provinces. A total of 24350 suicides deaths during four years of the study window were analyzed. We found an overall increase in the monthly suicide rate in Nepal with an average increase of 0.28 (CI: 0.12,0.45) suicide per 100,000 during the pandemic months. The increase in suicide rate was significant both among males (increase in rate = 0.26, CI: 0.02,0.50) and females (increase in rate = 0.30, CI: 0.18,0.43). The most striking increments in suicide rates were observed in June, July, and August 2020. The pattern of increased suicide rates faded away early on among males, but the effect was sustained for a longer duration among females. Sudurpaschim and Karnali provinces had the highest increase in suicide rates associated with the COVID-19 pandemic.

**Conclusions:**

The COVID-19 pandemic is associated with an increased suicide rate in Nepal. The findings may inform policymakers in designing appropriate public health responses to the pandemic that are considerate of the potential impact on mental health and suicide.

## Introduction

Coronavirus Disease 2019 (COVID-19) pandemic has affected peoples’ lives in unprecedented ways. The pandemic’s impact on virtually every domain of social life is well-established, including in the economy, education, public health, and health care. Psychologists and mental health experts have feared that the pandemic might escalate mental health problems across the countries [1–3]. An array of factors such as fear and anxiety of catching the virus, uncertainties about access to testing and medical care, the stress brought about by social distancing and lockdowns, loss of employment and growing medical costs, and social stigma of being infected could negatively affect mental health. Deteriorating mental health and psychosocial stresses could lead to an increase in suicidal ideation, and under more extreme scenarios, suicide deaths [4, 5].

Multiple studies conducted in different countries have reported higher levels of stress, anxiety, depression, insomnia, and substance abuse associated with the COVID-19 pandemic [6–9]. Studies exploring the relationship between the pandemic and mental health and suicidal behavior are mostly concentrated in developed countries. Given the differences in underlying contextual factors, the findings regarding the association between the pandemic and suicidal behavior from wealthy nations might not hold in low-income countries. There is limited data on the COVID-19 pandemic’s impact on suicidal behaviors in developing countries like Nepal and even rarer are the studies that specifically examine suicide mortality rates. Based on analysis of preliminary data, Pirikis et al. reported that the suicide rates did not increase in twenty-two upper-middle to high-income countries or regions [10]. In contrast, Tanaka and Okamoto found an increase in the suicide rate following an initial decline in Japan [11]. In Maryland US, although the overall suicide rate during the pandemic was similar to the pre-pandemic level, the African Americans had a significantly higher suicide mortality rate during the pandemic year [12]. A study conducted in India attributed the fear of COVID-19 as a prominent cause of suicide during the pandemic [13]. These findings indicate that the association between pandemic and suicide rate, if any, are highly heterogeneous. The effect of the pandemic on suicide could be country-specific and could depend on a multitude of factors including the prevalence of mental health comorbidities, the socio-economic setting, and the nature of the public health measures imposed by governments to contain the spread of the virus. Previous studies have documented the substantial variation in suicide rates by gender with a generally high suicide rate for males [14, 15] and by regions, even within the same country [16, 17]. Some empirical studies have reported mental health deterioration related to the pandemic to be higher in females than males [6, 18, 19]. This indicates a possible gender difference in the effects of the pandemic on suicide rates. Apart from being focused on high-income countries, most of the available studies on suicide rate and COVID-19 pandemic involve a narrower study window which makes it harder to disentangle the trend in suicide mortality from the well-known seasonality component in suicide rates [20] and also lack sufficient statistical power to detect the differences.

In this paper, we examine the association between the COVID-19 pandemic and suicide rates in Nepal. We hypothesize that the suicide rates might have increased during the pandemic in Nepal given the country’s poor public health infrastructure and imposition of strict lockdown orders and curfews to contain the spread of the virus. Furthermore, we seek to identify the impacts of the pandemic on suicides by gender and province. The understanding of the pandemic’s impact on suicide could be valuable in devising appropriate mental health programs and tailoring the current and future pandemic responses to minimize harm.

## Materials and methods

### Study setting and data

This is a retrospective study analyzing the suicide trends in Nepal over the study window from July 2017 to June 2021, which evaluates the effect of the COVID-19 pandemic on suicide rates. Nepal reported the first confirmed COVID-19 infection on January 23, 2020 [21]. The government responded to the contagion by imposing the first national lockdown on March 24, 2020 [22]. The first COVID-19 death was reported on May 14, 2020 [23]. In this paper, we define July 2017 to March 2020 as the pre-pandemic period (a total of 33 months) and April 2020 to June 2021 (a total of 15 months) as the pandemic period.

The monthly suicide death counts by gender for each of the seven provinces for the four Nepali fiscal years (Bikram Sambat (B.S.) 2074/75 to 2077/78) were obtained from Nepal Police headquarter, Kathmandu, Nepal. Every case of unnatural death in Nepal is investigated by the Police department as required by domestic law. The police determine the nature of death as a suicide based on the medical and autopsy reports. The pathway of determination and reporting of suicide in Nepal has been described in detail by Hagaman et al 2016 [24]. The seven provinces of Nepal are Province 1, Province 2, Bagmati, Gandaki, Lumbini, Karnali, and Sudurpaschim. The provinces are markedly different in socio-cultural and economic characteristics [25]. Since death counts were available by Nepali calendar months, deaths by Gregorian calendar months were calculated by assuming uniform death distribution within a given Nepali calendar month. (For example, the number of deaths in January 2021 is approximated by summing half of the deaths in the Nepali months of *Poush* and half of the deaths in *Magh*, B.S. 2077. The Gregorian calendar month starts at the approximate midpoint of the Nepali calendar month). The country-level yearly population estimate by gender (as of July 1 of each year) for 2017-2021 was obtained from United Nations Population Division, World Population Prospects 2019 [26]. The country-level monthly population estimates were computed by linear interpolation of the yearly population estimates. The 2017 population estimates for provinces were obtained from the Department of Health Services, Ministry of Health and Population, Nepal [27]. The province-level monthly population estimates were computed by applying the country-level population growth rate. The data of daily COVID-19 cases and deaths were obtained from John Hopkins University’s COVID-19 dashboard [28].

The Institutional Review Board (IRB) at Drexel University deemed that IRB approval is not necessary for the study as the study used aggregated, de-identified data.

### Statistical analysis

We calculated the suicide rates for each month during the study period by dividing the suicide deaths by the exposure population. Then we calculated incidence rate ratios (IRR) for each month during the pandemic period using the same month of 2019 as a reference. For example, the IRR for April 2020 is computed as a ratio of suicide rate in April 2020 to the suicide rate in April 2019.

Next, we employed linear regression models to estimate the effect of the pandemic on suicide rates. In our models, the monthly suicide rate per 100,000 population was the outcome variable, and the binary indicator variable (dummy) denoting whether a given month corresponds to the pandemic period was the primary exposure variable of interest. We also included month-fixed effects to adjust for the seasonality and year-fixed effects to adjust for the long-term trend in the suicide rates. The coefficient for the indicator variable would give an estimated average monthly change in suicide rates during the pandemic period, controlling for seasonal variation and long-term trends. We further extended our model to perform a month-by-month comparison of the suicide rates in the pandemic period to the average suicide rates in the pre-pandemic period. This was done by introducing an interaction term between the indicator variable and the month variable. We fit separate country-level models for the overall population, and for males and females. We operationalized the province-level models in a similar way. All the statistical analyses and data visualizations were performed in R (version: 4.0.2). The statistical significance was set at p<0.05.

## Results

Between July 2017 to June 2021, 24350 people committed suicide in Nepal, among which approximately 58% were male and 42% were female. The annual average suicide rate during four years of the study window was 21.3 per 100,000. The males had a higher suicide rate (26.9 per 100,000) than females (16.5 per 100,000). The number of suicides showed a substantial seasonal variation with monthly suicide numbers ranging from 368 to 569, 413 to 604, and 394 to 838 in 2018, 2019, and 2020 respectively. The lowest suicides were in January in all three years, whereas the highest suicides were in June in 2018 and 2019, and in July in 2020. During our study period, the highest proportion of the suicides were observed in Bagmati province (N = 5709; 23.4%), followed by Province 1 (N = 5161; 21.2%), Lumbini (N = 4757; 19.5%), Province 2 (N = 3053; 12.5%), Gandaki (N = 2369; 9.7%), Sudurpaschim (N = 2176; 8.9%), and Karnali (N = 1125, 4.6%).

**Fig 1:**
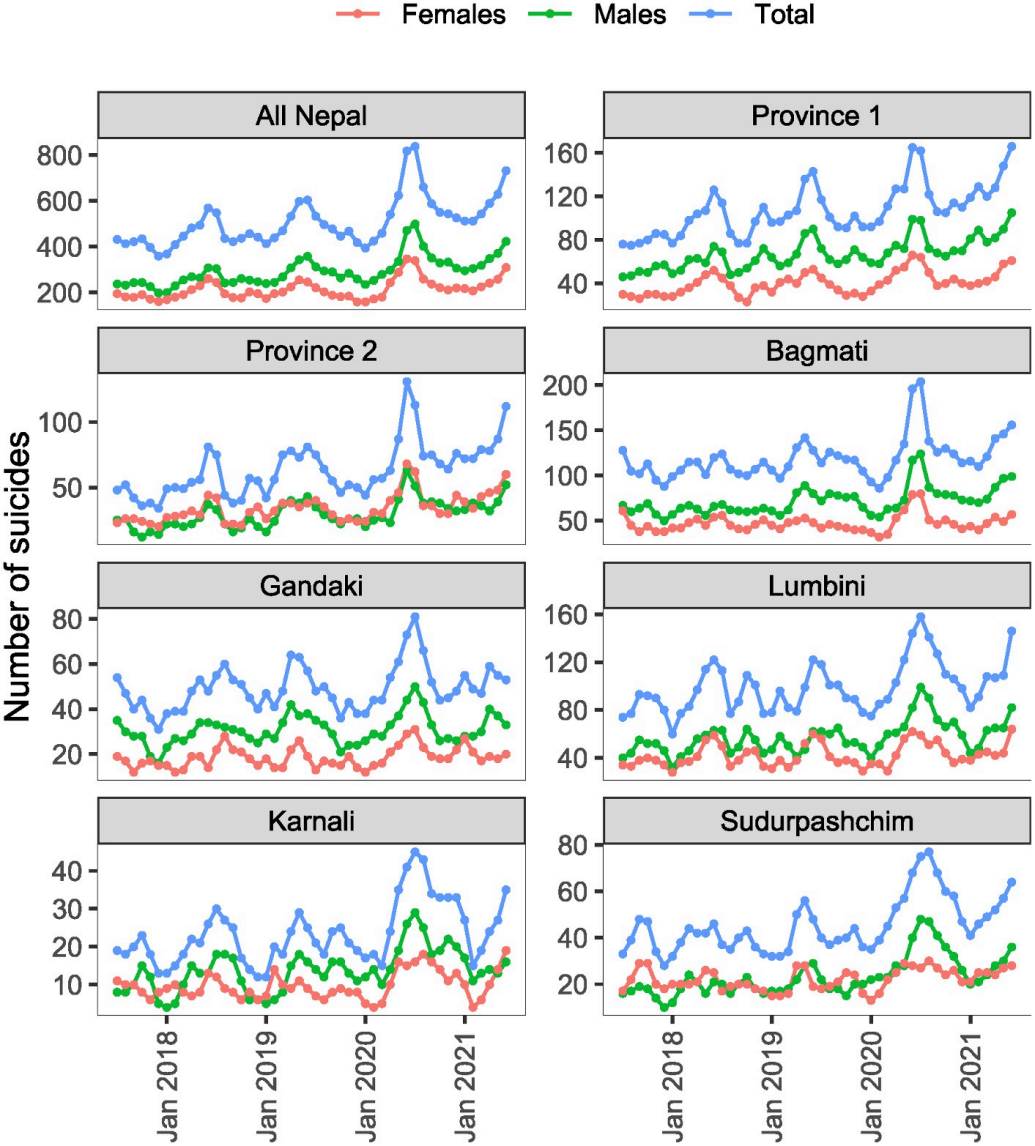
Monthly suicide deaths from July 2017 through June 2021 in Nepal by gender and province.

Table 1 presents the annual suicide counts and annual rates in 2018, 2019, and 2020, both at the country level and province level. There were 5509, 5898, and 6968 suicides in entire Nepal in 2018, 2019, and 2020 with corresponding annual rates of 19.6, 20.6, and 23.9 suicides per 100,000 population. The yearly increase in suicide rate from 2019 to 2020 is 16%, which is more than threefold higher than a yearly increase in the suicide rate from 2018 to 2019 (5%). Among the seven provinces, the suicide rate was highest in Province 1 and was lowest in Province 2 in all three years (Table 1). Monthly suicide death counts from July 2017 through June 2021, both at the country level and province level are shown in Fig 1.

**Table 1:** Country and province-level annual suicides numbers (annual suicide rates per 100,000 population).

Province	2018	2019	2020
Nepal	5509 (19.62)	5898 (20.63)	6968 (23.93)
Nepal, Males	3076 (24.09)	3463 (26.56)	4092 (30.68)
Nepal, Females	2433 (15.90)	2435 (15.65)	2876 (18.22)
Province 1	1157 (24.34)	1277 (26.40)	1438 (29.21)
Province 2	648 (10.95)	747 (12.40)	908 (14.81)
Bagmati	1311 (21.57)	1416 (22.89)	1561 (24.79)
Gandaki	569 (23.27)	580 (23.30)	650 (25.66)
Lumbini	1117 (23.03)	1133 (22.95)	1358 (27.03)
Karnali	240 (14.00)	256 (14.68)	371 (20.90)
Sudurpashchim	468 (16.85)	488 (17.26)	682 (23.70)

The incidence rate ratios (IRRs) of suicide for 12 pandemic months (April 2020 to March 2021) in comparison to the suicide rate in the same month in 2019 are presented in Table 2. All the pandemic months except April and May of 2020 and February and March of 2021 had a significantly higher suicide rate compared to the same months in 2019. In comparison to the same month in 2019, July 2020 had the highest increase in the suicide rate with an increment of 55% (IRR = 1.55, CI:1.39, 1.73), followed by June 2020 (IRR = 1.33, CI:1.20, 1.48).

**Table 2:** Monthly suicide counts, rates, and incidence rate ratio (IRR) in Nepal from 2018 to 2020.

Month	Number of suicides					Suicide rate per 100,000					IRR (95% CI)*
	2017	2018	2019	2020	2021	2017	2018	2019	2020	2021	2019 as reference
Jan		368	413	394	512	-	1.30	1.43	1.34	1.72	1.20 (1.05,1.37)
Feb		409	439	425	512	-	1.44	1.52	1.45	1.71	1.12 (0.99,1.27)
Mar		446	470	459	543	-	1.57	1.63	1.56	1.81	1.11 (0.98,1.26)
Apr		482	533	541	589	-	1.7	1.84	1.84	1.96	1.00 (0.89,1.13)
May		494	598	624	629	-	1.74	2.06	2.12	2.10	1.03 (0.92,1.15)
Jun		569	604	818	732	-	2.00	2.08	2.77	2.43	1.33 (1.20,1.48)
Jul	432	548	533	838	-	1.54	1.92	1.83	2.83	-	1.55 (1.39, 1.73)
Aug	413	436	497	661	-	1.47	1.52	1.71	2.23	-	1.30 (1.16,1.46)
Sep	422	422	478	588	-	1.50	1.47	1.64	1.98	-	1.21 (1.07,1.37)
Oct	435	437	446	550	-	1.54	1.52	1.53	1.85	-	1.21 (1.07,1.37)
Nov	397	457	468	544	-	1.41	1.59	1.60	1.83	-	1.14 (1.01,1.29)
Dec	359	442	418	526	-	1.27	1.54	1.43	1.76	-	1.23 (1.08,1.40)

*IRR calculated for pandemic months with 2019 as reference. April to December pandemic months are 2020 months, and January to March pandemic months are 2021 months.

**Fig 2:**
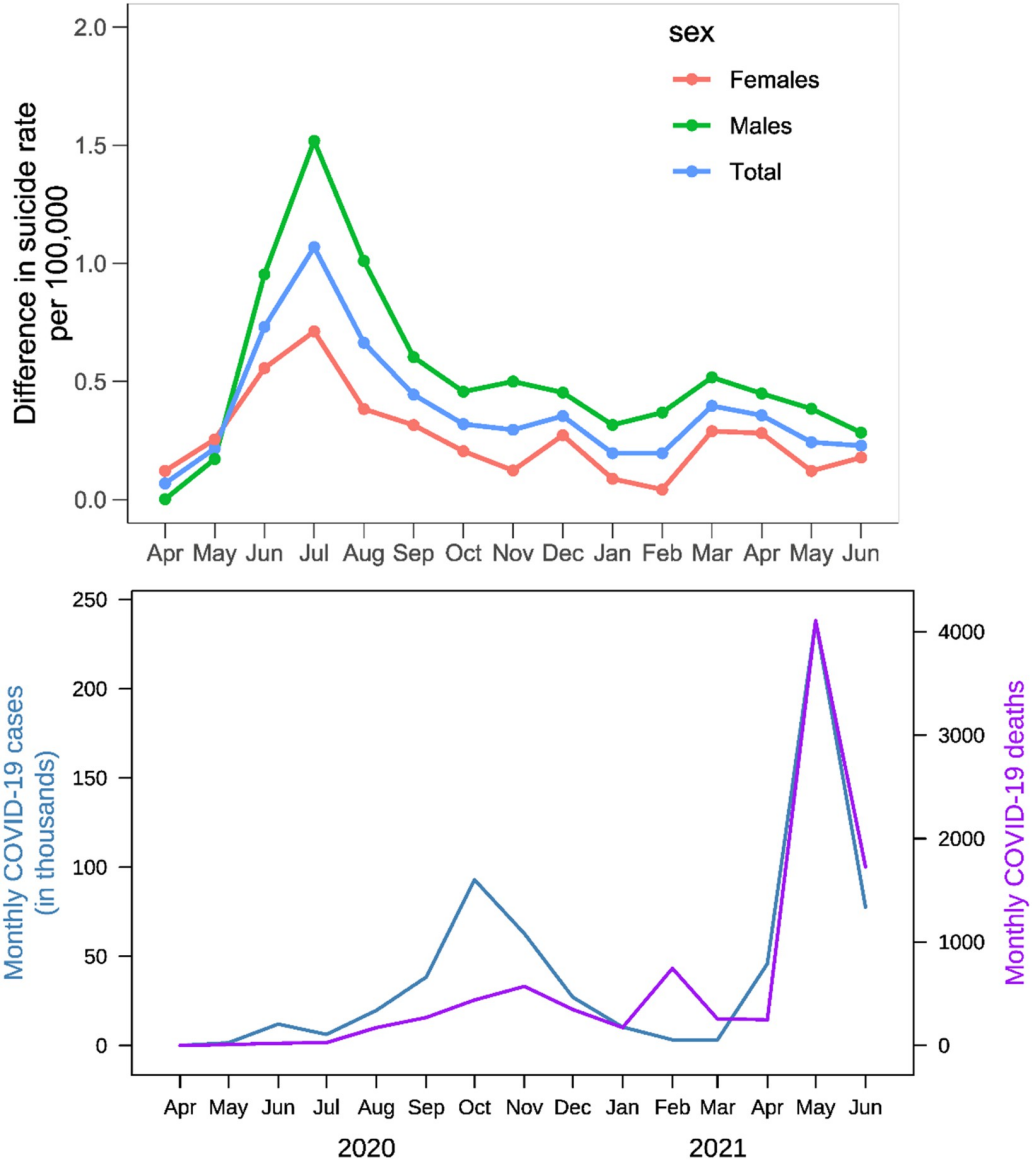
Unadjusted differences in monthly suicide rates in the pandemic months compared to the average rates of the same months in the immediate two pre-pandemic years (top panel) and monthly COVID-19 cases and deaths (bottom panel).

Fig 2 (top panel) presents the unadjusted differences in monthly suicide rates in the pandemic months compared to the average rates of the same months in the pre-pandemic period. The differences were close to zero in April 2020, and the highest differences were observed in July 2020. Fig 2 (bottom panel) presents trends of monthly COVID-19 cases and deaths over the pandemic period in Nepal. The estimated difference in the monthly suicide rates during the COVID-19 pandemic compared to the same months in the pre-pandemic period, adjusted for seasonality and overall temporal trend over the years, are presented in Table 3. Over the entire pandemic period, we saw an overall increase in monthly suicide rates with an average increase of 0.28 (CI: 0.12, 0.45) suicide per 100,000 population. The increase in suicide rate was significant both among males (increase in rate = 0.26, CI: 0.02, 0.50) and females (increase in rate = 0.30, CI: 0.18, 0.43). In the first two months of the pandemic (April and May of 2020), there was no effect on suicide rates. In those two months, both COVID-19 cases and deaths were low in Nepal Fig 2 (bottom panel).

**Table 3:** Adjusted estimated differences in country-level monthly suicide rates per 100,000 population during COVID-19 pandemic compared to same months in the pre-pandemic periods.

	Overall (Pooled Sex)		Males		Females	
Month	Estimate (95% CI)	P	Estimate (95% CI)	P	Estimate (95% CI)	P
All months*	0.28 (0.12,0.45)	0.002	0.26 (0.02,0.50)	0.039	0.30 (0.18,0.43)	<0.001
Apr,2020	-0.03 (-0.29,0.22)	0.782	-0.23 (-0.57,0.10)	0.165	0.13 (-0.11,0.37)	0.281
May,2020	0.11 (-0.14,0.37)	0.354	-0.06 (-0.40,0.27)	0.704	0.26 (0.02,0.50)	0.035
Jun,2020	0.63 (0.38,0.88)	<0.001	0.72 (0.38,1.06)	<0.001	0.56 (0.32,0.81)	<0.001
Jul, 2020	0.93 (0.69,1.18)	<0.001	1.21 (0.88,1.54)	<0.001	0.72 (0.49,0.96)	<0.001
Aug,2020	0.53 (0.28,0.77)	<0.001	0.70 (0.37,1.03)	<0.001	0.39 (0.16,0.63)	0.002
Sep,2020	0.31 (0.06,0.55)	0.016	0.29 (-0.04,0.62)	0.078	0.33 (0.09,0.56)	0.009
Oct,2020	0.18 (-0.06,0.43)	0.134	0.15 (-0.18,0.47)	0.367	0.21 (-0.02,0.45)	0.072
Nov,2020	0.16 (-0.09,0.40)	0.191	0.19 (-0.14,0.52)	0.245	0.13 (-0.10,0.37)	0.254
Dec,2020	0.22 (-0.03,0.46)	0.079	0.14 (-0.19,0.47)	0.378	0.28 (0.05,0.51)	0.021
Jan,2021	0.22 (-0.02,0.47)	0.075	0.14 (-0.19,0.47)	0.392	0.29 (0.05,0.52)	0.018
Feb,2021	0.11 (-0.14,0.35)	0.376	0.07 (-0.26,0.40)	0.648	0.13 (-0.11,0.37)	0.260
Mar,2021	0.09 (-0.15,0.34)	0.443	-0.03 (-0.36,0.30)	0.860	0.19 (-0.05,0.42)	0.113
Apr,2021	0.03 (-0.24,0.29)	0.839	-0.07 (-0.43,0.28)	0.673	0.10 (-0.16,0.36)	0.425
May,2021	0.03 (-0.24,0.29)	0.841	-0.02 (-0.38,0.34)	0.904	0.05 (-0.20,0.31)	0.662
Jun,2021	0.23 (-0.04,0.49)	0.092	0.13 (-0.23,0.49)	0.462	0.30 (0.04,0.56)	0.023

*Overall estimate for pandemic period vs pre-pandemic period.

The magnitude of association between the pandemic and suicide rates varied significantly over the months. The increase in monthly suicide rate (per 100,000) in the overall population was highest in July 2020 with an estimated increase of 0.93 (CI: 0.69, 1.18), followed by June 2020 (increase in rate = 0.63; CI: 0.38, 0.88), August 2020 (increase in rate = 0.53; CI: 0.28, 0.77), and September 2020 (increase in rate = 0.31; CI: 0.06, 0.55). Males had a significant increase in suicide rates in three 2020 pandemic months: June (increase in rate = 0.72; CI: 0.38, 1.06), July (increase in rate = 1.21; CI: 0.88,1.54), and August (increase in rate = 0.70; CI: 0.37, 1.03). Monthly suicide rates among females increased by 0.56 (0.32, 0.81), 0.72 (0.49, 0.96), 0.39 (0.16, 0.63) in June, July, and August of 2020, respectively. In addition to these three months, an increase in the suicide rate for females was also significant in the months of September and December of 2020 and January and June of 2021(Table 3).

Table 4 shows the adjusted changes in monthly suicide rates in each of the seven provinces in Nepal. The effects of the pandemic on suicide rates, both in terms of magnitude and duration of the effect, varied widely across the seven provinces. The highest increase in suicide rates averaged over the pandemic period was observed in Sudurpaschim province, followed by Karnali province, whereas Province 1 had the smallest increase. The increase in suicide rates was significant in July 2020 in all provinces. In addition, we found a significant increase in suicide rates in June 2020 in Province 2, Bagmati, and Gandaki provinces, and in August 2020 in Lumbini province. The effect was found to be spread over the longest duration in Sudurpaschim province where the increments were significant in June to September and November 2020, and in June 2021.

**Table 4:** Adjusted estimated difference in province-level monthly suicide rate per 100,000 population during COVID-19 pandemic compared to same months in the pre-pandemic periods.

Month	Estimate (95% CI)						
	Province 1	Province 2	Bagmati	Gandaki	Lumbini	Karnali	Sudurpaschim
All months*	0.16 (-0.04,0.36)	0.21(0.05,0.38)	0.30 (0.04,0.56)	0.21(0.07,0.50)	0.29 (0.04,0.55)	0.49 (0.18,0.80)	0.53 (0.27,0.78)
Apr,2020	0.14(-0.32,0.60)	-0.21 (-0.58,0.16)	-0.18 (-0.68,0.32)	-0.20 (-0.82,0.42)	0.17 (-0.41,0.75)	-0.06 (-0.62,0.49)	0.17(-0.25,0.60)
May,2020	-0.19 (-0.65,0.27)	0.21 (-0.17,0.58)	0.14 (-0.36,0.64)	-0.01 (-0.63,0.62)	0.17 (-0.41,0.74)	0.44 (-0.11,1.00)	0.21 (-0.22,0.63)
Jun,2020	0.31 (-0.15,0.77)	0.65 (0.27,1.02)	1.06 (0.56,1.56)	0.69 (0.07,1.31)	0.29 (-0.29,0.87)	0.75 (0.20,1.30)	0.66 (0.23,1.09)
Jul,2020	0.82 (0.37,1.27)	0.55 (0.19,0.92)	1.19 (0.70,1.68)	0.97 (0.36,1.58)	0.94 (0.37,1.51)	1.06 (0.52,1.60)	1.25 (0.83,1.67)
Aug,2020	0.32 (-0.13,0.77)	0.13 (-0.24,0.49)	0.30 (-0.19,0.79)	0.38 (-0.23,0.99)	0.93 (0.37,1.51)	1.08 (0.54,1.62)	1.30 (0.88,1.72)
Sep,2020	0.11 (-0.34,0.56)	0.28 (-0.08,0.65)	0.17 (-0.31,0.66)	0.09 (-0.52,0.69)	0.48 (-0.08,1.05)	0.46 (-0.08,1.00)	0.80 (0.38,1.21)
Oct,2020	0.07 (-0.38,0.52)	0.24 (-0.12,0.61)	0.21 (-0.28,0.70)	-0.14 (-0.74,0.47)	0.08 (-0.49,0.64)	0.48 (-0.06,1.02)	0.48 (0.06,0.90)
Nov,2020	-0.00 (-0.46,0.45)	0.04 (-0.33,0.40)	0.18 (-0.31,0.67)	0.00 (-0.61,0.61)	0.07 (-0.50,0.64)	0.71 (0.17,1.25)	0.61 (0.19,1.02)
Dec,2020	-0.10 (-0.55,0.35)	0.28 (-0.09,0.64)	0.08 (-0.40,0.57)	0.32 (-0.29,0.93)	0.22 (-0.35,0.79)	0.88 (0.34,1.42)	0.43 (0.01,0.85)
Jan,2021	0.23 (-0.22,0.68)	0.23 (-0.13,0.60)	0.17 (-0.32,0.65)	0.40 (-0.20,1.01)	0.06 (-0.51,0.62)	0.59 (0.05,1.13)	0.20 (-0.22,0.61)
Feb,2021	0.34 (-0.11,0.79)	0.08 (-0.28,0.45)	0.12 (-0.37,0.61)	0.16 (-0.45,0.76)	-0.07 (-0.64,0.49)	-0.30 (-0.84,0.24)	0.25 (-0.17,0.67)
Mar,2021	-0.07 (-0.52,0.38)	0.09 (-0.28,0.45)	0.11 (-0.38,0.59)	-0.02 (-0.62,0.59)	0.29 (-0.28,0.85)	-0.04 (-0.58,0.50)	0.19 (-0.23,0.60)
Apr,2021	-0.05 (-0.54,0.44)	-0.08 (-0.47,0.32)	0.15 (-0.38,0.68)	-0.09 (-0.75,0.57)	0.15 (-0.46,0.77)	-0.14 (-0.73,0.44)	0.09 (-0.37,0.54)
May,2021	0.02 (-0.47,0.51)	0.09 (-0.30,0.49)	0.25 (-0.28,0.78)	-0.32 (-0.99,0.34)	-0.19 (-0.80,0.43)	-0.09 (-0.68,0.50)	0.15 (-0.30,0.61)
Jun,2021	0.11 (-0.38,0.60)	0.21 (-0.18,0.61)	0.36 (-0.17,0.89)	-0.18 (-0.84,0.48)	0.22 (-0.40,0.84)	0.32 (-0.27,0.91)	0.46 (0.01,0.92)

*Overall estimate for pandemic period vs pre-pandemic period.

## Discussion

There is considerable heterogeneity in the findings that evaluate the effects of the pandemic on suicide mortality rates. In the case of severe acute respiratory syndrome (SARS) outbreak in 2003, suicide rates among the elderly population in Hong Kong appear to have increased [29]. Similarly, the deaths by suicide increased in the USA during the 1918–19 influenza pandemic [30]. We found a strong association between the COVID-19 pandemic and suicides in Nepal, after controlling for the long-term trend and seasonality in the suicide rates. Other studies have also reported a COVID-19 pandemic-related increase in suicide in some parts of South Asia [31, 32]. Our results are also consistent with the report of increased suicide and self-harm cases during the COVID-19 pandemic in one of the biggest teaching hospitals in Nepal [33]. Our results are different from a study that suggests the suicide rates have not increased during the COVID-19 pandemic in some high-income countries [10]. The differential effect of the pandemic on suicide rates between developed and low-income countries like Nepal is perhaps not surprising given the differences in economic resources, public health infrastructure, and awareness about mental health, and the outcomes could be worse in the resource-poor setting. Furthermore, the nature of the pandemic responses adopted in Nepal may be qualitatively different than many other countries since it took extraordinary measures of imposing curfews, lockdowns, and non-voluntary quarantine, mostly implemented by security forces with brute force.

Up to the second month of the pandemic, when the cumulative COVID-19 cases and deaths were below 1600 and 10 respectively, the suicide rates did not change significantly. The suicide rates began to increase starting in the third month of the pandemic and were significantly higher in June, July, and August of 2020. This corresponds to a period when there was a steep and rising trend of COVID-19 cases in Nepal. Contrary to studies that report a decrease in suicide immediately after the pandemic [11] or disaster [34, 35], we found a relatively early increase in the suicide rate. Unlike developed countries that provided financial stimulus packages at the early phase of the pandemic to relieve the distress of their population, Nepal did not offer such relief packages. Moreover, due to the shortages of ambulances and the ban on public and private transport during the lockdowns, time to reach the health care facilities may have been increased, resulting in the death of attempted-suicide patients who otherwise could have been saved [33]. In addition, the suspension of transportation and closure of hospitals for non-COVID 19 reasons may have created barriers to access mental health services.

We note that the months with the most increase in suicide rates correspond to the period of obsessive and panic media coverage of the Coronavirus in Nepal. Irresponsible media reportage about the virus, the conflicting messages by authorities, and the social stigma of being infected may have furthered the psychological stress, especially in the early months. As the pandemic progressed, people likely started to become habituated to the risks [36] and get used to a new normal. Apart from the panic-inducing media coverage of the COVID-19 infections and deaths, the quality of reporting on suicidal behavior and suicides is poor in South Asian countries, including Nepal [37]. Irresponsible media reporting and repeated exposure to suicide stories can prompt others to commit suicide [38–40]. The media (including the social media) coverage of suicide cases during the pandemic might have increased resulting in increased exposure to suicide triggers.

When looked at by gender, we found that males have a higher suicide mortality rate than females in Nepal, as in the case of many other countries [14]. This is also consistent with a published report from Nepal [41]. The increase in suicide rates was early on, and for a shorter period for males but the effect was sustained over a longer duration in females. This could be related to the gender differences in response to traumatic events and negative stimuli, where females are reported to experience sustained effects after traumatic exposure [42, 43]. However, future works should investigate the drivers behind this interesting phenomenon, perhaps in conjunction with differential psychosocial and economic contexts under which males and females operate [44].

Sudurpaschim and Karnali –two provinces that span the western part of the country—are found to have a substantially higher increment in the suicide rate during the pandemic period. These regions are reported to have a high poverty rate and low human development index [45], a summary measure of the status of human development that incorporates per capita gross national income, life expectancy, and level of education. Multiple studies have shown a positive association between poverty, unemployment and economic recession, and suicide [46–48]. Sudarpaschim and Karnali regions are also known to have a historically high proportion of seasonal migrant workers in India [49]. People in this region could have experienced additional distress as their migrant family members lost their jobs in India because of the business closure and lockdown imposed by India in the aftermath of the COVID-19 pandemic. Province 1 had the smallest increase in the suicide rate during the pandemic. To note, Province 1 has the highest human development index and relatively low poverty rates among seven provinces [45].

Our findings of increased suicide rates during the pandemic may have several policy implications. The governments and policymakers should be cautious of potential pathways through which the pandemic and subsequent public health responses to the pandemic could exacerbate the psychological and socio-economic drivers of suicide. Public health interventions to the pandemic, especially the severe ones like lockdowns and business closure, should incorporate economic safety nets and mental health service delivery mechanisms catered to geographic, socio-economic differences and needs. More epidemiological studies are needed to better understand the drivers of suicide in Nepal and the ways COVID-19 affected suicide in different subpopulations. The lack of appropriate data is one of the major bottlenecks for such studies in Nepal. A comprehensive suicide surveillance system to maintain detailed suicide statistics, perhaps within the jurisdiction of public health agencies rather than the law-enforcement agencies, is critically important.

### Limitations and strength

We used data obtained from the Nepal police in our study. Unlike in many countries where suicide data is maintained and reported by public health agencies, suicide statistics is handled by Nepal Police in Nepal. The agency lacks robust data recording systems and frequently relies on error-prone methods like paper-based recording and physical transfer of data [24]. This means that there could be some data quality issues. Despite this potential limitation, the data we used is the most comprehensive data currently available. The suicide data from Nepal police has been used in several studies [41, 50–52]. The suicide dataset we used was monthly aggregated data which means that we were not able to capture the variation in suicides in finer time intervals (e.g. days or weeks) or variations in individual-level characteristics such as age or reasons of suicide. Furthermore, the analysis was only adjusted for seasonality, and overall temporal trends in suicide rates, and we could not account for the potential factors specific to pandemic months other than COVID-19 that could have influenced the suicide rates. To our knowledge, this is the first study of its kind analyzing the suicide trends in Nepal in relation to the COVID-19 pandemic in the overall population, and by gender and province.

## Conclusion

The suicide rates significantly increased in Nepal in the early months of the COVID-19 pandemic, even after controlling for seasonality and longer-term trends of suicide rate. The effects varied by gender and province. The increment in suicide rates among males was limited to the first few months into the pandemic while we observed an extended impact over a longer period among females. We found a much bigger impact of the pandemic in Sudurpaschim and Karnali provinces. Epidemiological studies are warranted to better understand the drivers of overall high suicide rates and the gender and geographic differences in the suicide rates. Future research should explore the reasons behind the differential impacts of the COVID-19 pandemic in different provinces and gender groups.

## References

[1] Pfefferbaum B, North CS. Mental health and the Covid-19 pandemic. New England Journal of Medicine. 2020;383(6):510–512. doi: 10.1056/NEJMp2008017 32283003

[2] World Health Organization. Mental health and psychosocial considerations during the COVID-19 outbreak, 18 March 2020. World Health Organization; 2020.

[3] Holmes EA, O’Connor RC, Perry VH, Tracey I, Wessely S, Arseneault L, et al. Multidisciplinary research priorities for the COVID-19 pandemic: a call for action for mental health science. The Lancet Psychiatry. 2020;7(6):547–560. doi: 10.1016/S2215-0366(20)30168-1 32304649 PMC7159850

[4] Sher L. Psychiatric disorders and suicide in the COVID-19 era. QJM: An International Journal of Medicine. 2020;113(8):527–528. doi: 10.1093/qjmed/hcaa204 32569376 PMC7337853

[5] Reger MA, Stanley IH, Joiner TE. Suicide mortality and coronavirus disease 2019—a perfect storm? JAMA psychiatry. 2020;77(11):1093–1094. 32275300 10.1001/jamapsychiatry.2020.1060

[6] Pieh C, Budimir S, Probst T. The effect of age, gender, income, work, and physical activity on mental health during coronavirus disease (COVID-19) lockdown in Austria. Journal of psychosomatic research. 2020;136:110186. doi: 10.1016/j.jpsychores.2020.110186 32682159 PMC7832650

[7] Grover S, Sahoo S, Mehra A, Avasthi A, Tripathi A, Subramanyan A, et al. Psychological impact of COVID-19 lockdown: An online survey from India. Indian Journal of Psychiatry. 2020;62(4):354. doi: 10.4103/psychiatry.IndianJPsychiatry_427_20 33165368 PMC7597717

[8] Czeisler M, Lane RI, Petrosky E, Wiley JF, Christensen A, Njai R, et al. Mental health, substance use, and suicidal ideation during the COVID-19 pandemic—United States, June 24–30, 2020. Morbidity and Mortality Weekly Report. 2020;69(32):1049. doi: 10.15585/mmwr.mm6932a1 32790653 PMC7440121

[9] Dong L, Bouey J. Public Mental Health Crisis during COVID-19 Pandemic, China. Emerg Infect Dis. 2020;26(7):1616–1618. doi: 10.3201/eid2607.200407 32202993 PMC7323564

[10] Pirkis J, John A, Shin S, DelPozo-Banos M, Arya V, Analuisa-Aguilar P, et al. Suicide trends in the early months of the COVID-19 pandemic: an interrupted time-series analysis of preliminary data from 21 countries. The Lancet Psychiatry. 2021;8(7):579–588. doi: 10.1016/S2215-0366(21)00091-2 33862016 PMC9188435

[11] Tanaka T, Okamoto S. Increase in suicide following an initial decline during the COVID-19 pandemic in Japan. Nature human behaviour. 2021;5(2):229–238. doi: 10.1038/s41562-020-01042-z 33452498

[12] Bray MJC, Daneshvari NO, Radhakrishnan I, Cubbage J, Eagle M, Southall P, et al. Racial differences in statewide suicide mortality trends in Maryland during the coronavirus disease 2019 (COVID-19) pandemic. JAMA psychiatry. 2021;78(4):444–447. doi: 10.1001/jamapsychiatry.2020.3938 33325985 PMC7745133

[13] Dsouza DD, Quadros S, Hyderabadwala ZJ, Mamun MA. Aggregated COVID-19 suicide incidences in India: Fear of COVID-19 infection is the prominent causative factor. Psychiatry research. 2020;290:113145. doi: 10.1016/j.psychres.2020.113145 32544650 PMC7832713

[14] Khazaei S, Armanmehr V, Nematollahi S, Rezaeian S, Khazaei S. Suicide rate in relation to the Human Development Index and other health related factors: A global ecological study from 91 countries. Journal of epidemiology and global health. 2017;7(2):131–134. doi: 10.1016/j.jegh.2016.12.002 28188120 PMC7320427

[15] Värnik P. Suicide in the world. International journal of environmental research and public health. 2012;9(3):760–771. doi: 10.3390/ijerph9030760 22690161 PMC3367275

[16] Jagodic HK, Agius M, Pregelj P. Inter-regional variations in suicide rates. Psychiatr Danub. 2012;24(Suppl 1):S82–5. 22945194

[17] Middleton N, Gunnell D, Frankel S, Whitley E, Dorling D. Urban–rural differences in suicide trends in young adults: England and Wales, 1981–1998. Social science and medicine. 2003;57(7):1183–1194. doi: 10.1016/S0277-9536(02)00496-3 12899903

[18] Proto E, Quintana-Domeque C. COVID-19 and mental health deterioration by ethnicity and gender in the UK. PloS one. 2021;16(1):e0244419. doi: 10.1371/journal.pone.0244419 33406085 PMC7787387

[19] Moghanibashi-Mansourieh A. Assessing the anxiety level of Iranian general population during COVID-19 outbreak. Asian journal of psychiatry. 2020;51:102076. doi: 10.1016/j.ajp.2020.102076 32334409 PMC7165107

[20] Christodoulou C, Douzenis A, Papadopoulos FC, Papadopoulou A, Bouras G, Gournellis R, et al. Suicide and seasonality. Acta Psychiatrica Scandinavica. 2012;125(2):127–146. doi: 10.1111/j.1600-0447.2011.01750.x 21838741

[21] Shrestha R, Shrestha S, Khanal P, Kc B. Nepal’s first case of COVID-19 and public health response. Journal of Travel Medicine. 2020;27(3):taaa024. doi: 10.1093/jtm/taaa024 32104884 PMC7107523

[22] Nepal goes into lockdown for a week. Nepali Times. 2020. Available from: https://www.nepalitimes.com/banner/nepal-goes-into-lockdown-for-a-week/.

[23] Shrestha A, Bhushal N, Shrestha A, Tamrakar D, Adhikari P, Shrestha P, et al. First reported death of a postpartum woman due to coronavirus disease 2019 in nepal: a lesson learnt. Kathmandu University Medical Journal. 2020;18(2):117–119. doi: 10.3126/kumj.v18i2.33074 33605254

[24] Hagaman AK, Maharjan U, Kohrt BA. Suicide surveillance and health systems in Nepal: a qualitative and social network analysis. International journal of mental health systems. 2016;10(1):1–19. doi: 10.1186/s13033-016-0073-7 27274355 PMC4895957

[25] Nepali S, Ghale S, Hachhethu K. Federal Nepal. The Provinces (Socio-Cultural Profiles of the Seven Provinces). Governance Facility. 2018.

[26] World Population Prospects 2019. United Nations, Department of Economic and Social Affairs. 2019. Available from: https://population.un.org/wpp/.

[27] Department of Health Services. Ministry of Health and Population, Nepal. 2019. Available from: https://dohs.gov.np/wp-content/uploads/2019/07/HMIS-Database.2074_75_by_Local_Government.xlsx.

[28] COVID-19 Data Repository by the Center for Systems Science and Engineering (CSSE) at Johns Hopkins University. 2021 Available from: https://github.com/CSSEGISandData/COVID-19.

[29] Chan SMS, Chiu FKH, Lam CWL, Leung PYV, Conwell Y. Elderly suicide and the 2003 SARS epidemic in Hong Kong. International Journal of Geriatric Psychiatry: A journal of the psychiatry of late life and allied sciences. 2006;21(2):113–118. doi: 10.1002/gps.1432 16416469

[30] Wasserman IM. The impact of epidemic, war, prohibition and media on suicide: United States, 1910–1920. Suicide and Life Threatening Behavior. 1992;22(2):240–254. 1626335

[31] Mamun MA, Ullah I. COVID-19 suicides in Pakistan, dying off not COVID-19 fear but poverty?–The forthcoming economic challenges for a developing country. Brain, behavior, and immunity. 2020;87:163. doi: 10.1016/j.bbi.2020.05.028 32407859 PMC7212955

[32] Bhuiyan AI, Sakib N, Pakpour AH, Griffiths MD, Mamun MA. COVID-19-related suicides in Bangladesh due to lockdown and economic factors: case study evidence from media reports. International Journal of Mental Health and Addiction. 2020; p. 1–6. doi: 10.1007/s11469-020-00307-y 32427168 PMC7228428

[33] Shrestha R, Siwakoti S, Singh S, Shrestha AP. Impact of the COVID-19 pandemic on suicide and self-harm among patients presenting to the emergency department of a teaching hospital in Nepal. PLoS one. 2021;16(4):e0250706. doi: 10.1371/journal.pone.0250706 33930044 PMC8087018

[34] Kõlves K, Kõlves KE, De Leo D. Natural disasters and suicidal behaviours: a systematic literature review. Journal of affective disorders. 2013;146(1):1–14. doi: 10.1016/j.jad.2012.07.037 22917940

[35] Orui M, Sato Y, Tazaki K, Kawamura I, Harada S, and Hayashi M. Delayed increase in male suicide rates in tsunami disaster-stricken areas following the great east japan earthquake: a three-year follow-up study in Miyagi Prefecture. Tohoku J Exp Med. 2015;235:215–222 doi: 10.1620/tjem.235.215 25765170

[36] Slovic P. Perception of risk. Science. 1987;236(4799):280–285. doi: 10.1126/science.3563507 3563507

[37] Arafat SY, Kar SK, Marthoenis M, Cherian AV, Vimala L, Kabir R. Quality of media reporting of suicidal behaviors in South-East Asia. Neurology, Psychiatry and Brain Research. 2020;37:21–26. doi: 10.1016/j.npbr.2020.05.007

[38] Phillips DP. The influence of suggestion on suicide: Substantive and theoretical implications of the Werther effect. American sociological review. 1974; p. 340–354. doi: 10.2307/2094294 11630757

[39] Niederkrotenthaler T, Braun M, Pirkis J, Till B, Stack S, Sinyor M, et al. Association between suicide reporting in the media and suicide: systematic review and meta-analysis. Bmj. 2020;368. doi: 10.1136/bmj.m575 32188637 PMC7190013

[40] Niederkrotenthaler T, Voracek M, Herberth A, Till B, Strauss M, Etzersdorfer E, et al. Role of media reports in completed and prevented suicide: Werther v. Papageno effects. The British Journal of Psychiatry. 2010;197(3):234–243. doi: 10.1192/bjp.bp.109.074633 20807970

[41] Hagaman AK, Khadka S, Lohani S, Kohrt B. Suicide in Nepal: a modified psychological autopsy investigation from randomly selected police cases between 2013 and 2015. Social psychiatry and psychiatric epidemiology. 2017;52(12):1483–1494. doi: 10.1007/s00127-017-1433-6 28856382 PMC5705471

[42] Andreano JM, Dickerson BC, Barrett LF. Sex differences in the persistence of the amygdala response to negative material. Social Cognitive and Affective Neuroscience. 2014;9(9):1388–1394. doi: 10.1093/scan/nst127 24036962 PMC4158377

[43] Hu J, Feng B, Zhu Y, Wang W, Xie J, Zheng X. Gender differences in PTSD: susceptibility and resilience. Gender differences in different contexts InTech(ed) Aida Alvinius. 2017; p. 21–42.

[44] Thapaliya S, Sharma P, Upadhyaya K. Suicide and self harm in Nepal: A scoping review. Asian journal of psychiatry. 2018;32:20–26. doi: 10.1016/j.ajp.2017.11.018 29202423

[45] Dhungel S. Provincial comparison of development status in Nepal: an analysis of human development trend for 1996 to 2026. Journal of Management and Development Studies. 2018;28:53–68. doi: 10.3126/jmds.v28i0.24958

[46] Oyesanya M, Lopez-Morinigo J, Dutta R. Systematic review of suicide in economic recession. World journal of psychiatry. 2015;5(2):243. doi: 10.5498/wjp.v5.i2.243 26110126 PMC4473496

[47] Kawohl W, Nordt C. COVID-19, unemployment, and suicide. The Lancet Psychiatry. 2020;7(5):389–390. doi: 10.1016/S2215-0366(20)30141-3 32353269 PMC7185950

[48] Iemmi V, Bantjes J, Coast E, Channer K, Leone T, McDaid D, et al. Suicide and poverty in low-income and middle-income countries: a systematic review. The Lancet Psychiatry. 2016;3(8):774–783. doi: 10.1016/S2215-0366(16)30066-9 27475770

[49] Thieme S, Müller-Böker U. Financial self-help associations among Far West Nepalese labor migrants in Delhi, India. Asian and Pacific Migration Journal. 2004;13(3):339–361. doi: 10.1177/011719680401300303

[50] Mishra N, Shrestha D, Poudyal RB. Retrospective study of suicide among children and young adults. Journal of Nepal Paediatric Society. 2013;33(2):110–116. doi: 10.3126/jnps.v33i2.7512

[51] Acharya SR, Shin YC, Moon DH. COVID-19 outbreak and suicides in Nepal: urgency of immediate action. International journal of social psychiatry. 2020:0020764020963150. 32985316 10.1177/0020764020963150

[52] Singh R, Baral KP, Mahato S. An urgent call for measures to fight against increasing suicides during COVID-19 pandemic in Nepal. Asian journal of psychiatry. 2020;54:102259. doi: 10.1016/j.ajp.2020.102259 32619837 PMC7305514

